# Incidence, Treatment, and Survival of Patients With T-Cell Lymphoma, T-Cell Large Granular Leukemia, and Concomitant Plasma Cell Dyscrasias

**DOI:** 10.3389/fonc.2022.858426

**Published:** 2022-04-29

**Authors:** Zachary Braunstein, Eric McLaughlin, Miguel Ruiz, Lai Wei, Naresh Bumma, Don Benson, Srinivas Devarakonda, Maria Chaudhry, Abdullah Khan, Francesca Cottini, Walter Hanel, Robert Baiocchi, Catherine Chung, Daniel Addison, Nina Couette, Alexa Meara, Wael Jarjour, Pierluigi Porcu, Anjali Mishra, John C. Reneau, Ashley E. Rosko, Jonathan E. Brammer

**Affiliations:** ^1^ Department of Internal Medicine, The Ohio State University Wexner Medical Center, Columbus, OH, United States; ^2^ Center for Biostatistics, Department of Biomedical Informatics, The Ohio State University, Columbus, OH, United States; ^3^ Division of Hematology, Department of Internal Medicine, James Comprehensive Cancer Center, The Ohio State University, Columbus, OH, United States; ^4^ Division of Hematology, George Washington Cancer Center, George Washington University, Washington, DC, United States; ^5^ Department of Dermatology, The Ohio State University Wexner Medical Center, Columbus, OH, United States; ^6^ Cardio-Oncology Program, Division of Cardiology, Department of Internal Medicine, The Ohio State University Wexner Medical Center, Columbus, OH, United States; ^7^ Division of Rheumatology, Department of Internal Medicine, The Ohio State University Wexner Medical Center, Columbus, OH, United States; ^8^ Division of Hematologic Malignancies and Hematopoietic Stem Cell Transplantation, Department of Medical Oncology and Department of Cancer Biology, Sydney Kimmel Cancer Center, Thomas Jefferson University, Philadelphia, PA, United States; ^9^ Division of Hematologic Malignancies and Hematopoietic Stem Cell Transplantation, Department of Medical Oncology, Sydney Kimmel Cancer Center, Thomas Jefferson University, Philadelphia, PA, United States

**Keywords:** T cell, CTCL, T-LGL, PTCL, MGUS, multiple myeloma, plasma cell dyscrasia, survival

## Abstract

T-Cell malignancies are a group of heterogeneous disorders composed of primary cutaneous T-cell lymphomas (CTCLs), peripheral T-cell lymphomas (PTCLs), and T-cell leukemias, including T-cell large granular lymphocytic leukemia (T-LGLL). Cases of patients with combined T-cell malignancies and plasma cell dyscrasias (PCD) are reported in the literature, but these are mostly limited to case reports or small case series with <10 patients. Here, we described the clinical course of 26 patients and report baseline characteristics and clinical outcomes including overall survival (OS), progression-free survival (PFS), and objective response rates (ORRs) in this unique population. There was no survival difference in patients with CTCL or T-LGLL and concomitant PCD when treated with standard therapy directed at the T-cell malignancy when compared to historical controls. However, patients with PTCL and concomitant PCD had significantly inferior outcomes with rapid progression and worse OS and PFS at 1.7 years (p=0.006) and 4.8 months (p=0.08), respectively, when compared to historical controls for patients with PTCL, although the limited number of patients included in this analysis precludes drawing definitive conclusions. Treatment directed at the T-cell malignancy resulted in the eradication of the PCD clone in multiple patients (15.4%) including one with multiple myeloma (MM) who experienced a complete response after starting therapy directed at the T-cell malignancy. For patients with T-cell malignancies and concomitant PCD, treatment with standard T-cell-directed therapies is recommended based on this analysis with continued follow-up and monitoring of the concomitant PCD. Further studies are needed to definitively elucidate the increased risk of relapse in patients with PTCL and concomitant PCD, and larger, multi-center cohorts are needed to validate these findings across T-cell malignancies and PCDs.

## Introduction

T-Cell malignancies are a group of heterogeneous disorders, including cutaneous T-cell lymphomas (CTCLs), peripheral T-cell lymphomas (PTCLs), and T-cell leukemias, such as T-cell large granular lymphocytic leukemia (T-LGLL). T-LGLL is an incurable mature T-cell leukemia characterized by the abnormal clonal proliferation of CD3+/CD5/DimCD8+/CD57+T cells (cytotoxic T-lymphocytes, CTLs) which can result in severe neutropenia, transfusion-dependent anemia, and marrow failure. Patients require frequent therapy, with recurrent relapses and overall response rates (ORRs) approximately 40% (1), although overall survival is >10 years in most patients (2–4). PTCL, of which the primary subtypes include anaplastic large cell lymphoma (ALCL) (25%), angioimmunoblastic T-cell lymphoma (33%), and PTCL-NOS (40%), are aggressive lymphomas with poor long-term survival of 35% at 5 years outside of ALK+ ALCL (5–7). CTCL, of which the most common variety is mycosis fungoides (MF), is a chronic dermatological condition that often requires frequent, sequential therapies (8). A deeper understanding of these disorders and associated prognostic and contributing factors is essential to improve outcomes in these rare diseases.

Sporadic cases of patients with combined T-cell malignancies and plasma cell dyscrasias (PCD) have been reported in the literature. These include small series and case reports of patients with T-cell lymphomas or T-LGLL with concomitant multiple myeloma (MM), monoclonal gammopathy of undetermined significance (MGUS), and other PCDs (9–12). While the most commonly observed association is with T-LGLL, there are case reports of other T-cell malignancies including AITL and PTCL-NOS with MM. Due to the rarity of these diseases, little is known about the pathophysiology, or clinical significance of these findings, and whether clinical or disease-related outcomes are impacted. Most commonly, T-LGLL with concomitant PCD or MM has been described. These include a few singular case studies of patients that have concomitant T-LGLL and PCD, including MM and even amyloidosis (9–15). There is only one case series with >10 patients, which is mainly descriptive in nature (16), while another study with six patients is also descriptive but does start to explore the potential link between the two diseases (17). The exact mechanism of interrelation between these disorders is not well known, but there are some postulations about how they link together, particularly in the newly describe T-follicular helper-type (TFH) lymphomas, as TFH cells regulate B cells, and there is a clear association with B-cell activation in these lymphomas, including plasma cells (18, 19). Furthermore, the clinical significance, including response and survival outcomes, of these coincident disorders remains unknown.

The purpose of this study was to explore the prognostic factors and outcomes of patients who have concomitant TCL or T-LGLL and PCD. Specifically, we investigated survival outcomes in patients with concomitant T-cell malignancies and PCD and evaluate the prognostic impact on treatment response and survival in this unique population.

## Patients and Methods

### Patients

This study is a retrospective review of all patients diagnosed at the OSU James Cancer Center (OSUCCC) with a concomitant T-cell malignancy and PCD between January 1, 2011 and October 1, 2021. Patients were identified from The Ohio State University (OSU) lymphoma database, OSU MM database, and OSU T-LGLL registry. This study was approved by the Institutional Review Board at OSU.

### Diagnosis of T-Cell Malignancies

All diagnoses for T-cell malignancies were made based on the 2016 World Health Organization (WHO) criteria. Given the difficulty in diagnosing T-LGLL, we included specific criteria for the diagnosis of T-LGLL, adapted from the 2016 WHO criteria, recently utilized in the ECOG5998 trial and recent studies (4, 20, 21). T-LGLL diagnosis required the presence of a monoclonal T-cell receptor (TCR) and a CD3+CD8+ population on flow cytometry ≥500 cells/mm (3). A monoclonal T-cell receptor was positive if detected by TCR polymerase chain reaction (PCR) or by restriction of TCR Vbeta noted on flow cytometry. For patients diagnosed with a clonal TCR by flow cytometry, a panel of 30 TCR Vbeta rearrangements was used with positivity considered if one or more clone was detected in 10% of events or greater as previously described (22).

### Diagnosis of Plasma Cell Dyscrasias

The diagnoses for PCD were made based on the 2016 WHO criteria or the revised International Myeloma Working Group (IMWG) criteria. The diagnosis of MGUS was made if a patient had the presence of a monoclonal protein, <10% clonal plasma cells on bone marrow biopsy, and no other features of MM, such as anemia, renal dysfunction, or bone disease (23). The diagnosis of MM was made in patients with the presence of a monoclonal protein and an abnormal free light chain ratio, and clinical features of MM including anemia, renal dysfunction, and/or bone disease or a myeloma defining event such as ≥60% clonal plasma cells on bone marrow examination, more than one focal lesion on MRI ≥ 5mm, or serum-free light chain ration ≥100 (24, 25).

### Follow-up and Response Assessment

All patients with T-LGLL/TCL were followed from 1998 to 2018 in the T-cell malignancy clinic at the OSUCCC, staffed by a dedicated T-cell physician. The workflow, diagnostic, and treatment approach were thus standardized over time. On treatment, patients were typically seen in the clinic every 2–3 months. Patients off treatment, or on observation, were typically followed every 6 months to 1 year. Treatment regimens varied by patient based upon the clinical scenario. Patients were also seen by a dedicated plasma cell physician in the Plasma Cell Clinic at the OSUCCC. Patients with no high-risk features were typically seen annually for MGUS. Patients with smoldering disease were seen every 3–4 months depending on clinical characteristics, and patients with active myeloma are seen monthly or sooner as needed. Treatment regimens were varied based on the clinical scenario. For patients with nodal PTCL, responses were determined *via* Lugano criteria (26). For patients with T-LGLL, responses were based off of the modified ECOG5998 criteria, as reported in a recent study (4) and a recent prospective trial in T-LGLL (27), and were assessed by the investigators. At least 4 months of treatment were needed to assess for response (**Supplementary Table S1**). For patients with CTCL, response was determined based on the criteria for consensus statement of Olsen et al. (28) For patients with MM, response criteria were determined by the International Myeloma Working Group Uniform Response Criteria for CR, namely, very good partial response (VGPR), PR, stable disease (SD), and no response (NR) (25, 29).

### Statistical Analysis

Baseline demographics and clinical characteristics were reported using summary statistics for the overall sample and by the type of malignancy. Overall survival (OS) was assessed as time from T-cell malignancy diagnosis until death or censoring. Progression-free survival (PFS) was assessed as the time from T-cell malignancy diagnosis until progression, death, or censoring. Patients without OS or PFS events were censored at last follow-up. Median OS and median PFS, along with the 95% confidence intervals, were calculated using Kaplan–Meier methods for the overall sample and by malignancy type. Survival curves were compared among the type of monoclonal protein using the log-rank test. Response to treatment was also reported for the overall sample and by malignancy type. All analyses were performed using SAS version 9.4 (SAS Institute Inc., Cary, NC, USA).

## Results

### Entire Cohort

A total of 26 patients with confirmed concomitant T-cell malignancy and PCD were included in this analysis. Full patient baseline characteristics are seen in **Table 1**. The median age at T-cell malignancy diagnosis was 63 (range, 39–82; SD, 10.9) years, and the median age at PCD diagnosis was 64 (30–82, 12.3) years; 65% (n = 17) of patients were male, and 96% (n = 25) were Caucasian. Ten (39%) of the patients presented with their T-cell malignancy first, and 10 (39%) presented with their PCD first, while 19% (n = 5) had a concurrent diagnosis, and for one patient (4%), this was unknown. The most common concurrent T-cell malignancy was T-LGLL (n = 14, 54%), followed by CTCL (n = 6, 23%) and PTCL (n = 6, 23%). The most common PCD was MGUS (n = 13, 50%), followed by MM (n = 8, 31%) and plasmacytosis (n = 2, 8%). Plasmacytoma, lymphoplasmacytic lymphoma (LPL), and a kappa light chain-predominant plasma cell proliferation were seen in one patient (4%) each. The plasmacytosis diagnosis and kappa light chain-predominant plasma cell proliferation diagnosis was given to the patients by their treating physician and included as such in this study. On review, based on IMWG criteria, these patients would likely meet diagnostic criteria for MGUS. Overall, 16/26 (62%) patients were treated for their T-cell malignancy frontline, while 9/26 (35%) were treated for their PCD frontline, and one patient (4%) did not receive treatment for either disease.

**Table 1:** Baseline characteristics for all patients.

Variable	Total (%) (n=26)
Age at T-cell diagnosis, mean (SD)	63.2 (10.9)
Age at PCD diagnosis, mean (SD)	63.7 (12.3)
***Sex***	
Male	17 (65.4)
Female	9 (34.6)
***Race***	
Caucasian	25 (96.2)
African American	1 (3.8)
***Primary Presenting Malignancy***	
T-Cell Malignancy	10 (38.5)
PCD	10 (38.5)
Concurrent Diagnosis	5 (19.2)
Unknown	1 (3.8)
***T-Cell Malignancy***	
T-LGLL	14 (53.8)
PTCL	6 (23.1)
-PTCL-NOS	4 (15.4)
-AITL	2 (7.7)
CTCL	6 (23.1)
***Plasma Cell Dyscrasia***	
MGUS	13 (50.0)
MM	8 (30.1)
Plasmacytosis	2 (7.7)
Plasmacytoma	1 (3.8)
LPL	1 (3.8)
kappa light chain-predominant plasma cell proliferation	1 (3.8)
***Monoclonal Protein-Light Chain***	
IgA-L	1 (3.8)
IgA-Unk	3 (11.5)
IgG-K	8 (30.8)
IgG-L	3 (11.5)
IgM-K	2 (7.7)
IgM-L	2 (7.7)
N/A-K	2 (7.7)
N/A-L	2 (7.7)
None Detected	2 (7.7)
Unknown	1 (3.8)
Percent bone marrow plasma cells at PCD diagnosis, median (SD; range)	5 (23.0; 0.5–80.0)
M-protein quantity at diagnosis (mg/dl), median (SD; range)	533 (1,564; 15.0–6,042.0)
Serum free light chain ratio at PCD diagnosis, median (SD; range)	7.1 (38.2; 1.1–130.7)
***ISS Staging For PCD***	
1	4 (15.4)
2	2 (7.7)
3	3 (11.5)
N/A	17 (65.4)
***First-Line T-Cell Malignancy Therapy***	16/26* (61.5)
Methotrexate	5 (31.3)
Cyclophosphamide	1 (6.3)
Cyclosporine	3 (18.8)
CHOP	3 (18.8)
EPOCH	2 (12.5)
Skin Directed Therapy	2 (12.5)
***First-Line PCD Therapy***	9/26* (34.6)
Bortezomib/Lenalidomide/Dexamethasone	4 (44.4)
Bortezomib/Dexamethasone	1 (11.1)
Cyclophosphamide/Bortezomib/Dexamethasone	1 (11.1)
Doxorubicin/Vincristine/Dexamethasone	1 (11.1)
Daratumumab/Lenalidomide	1 (11.1)
IFRT	1 (11.1)

*One patient has not received treatment for either disease.

AITL, angioimmunoblastic T-cell lymphoma; Alk Phos, alkaline phosphatase; CHOEP, Cyclophosphamide, Doxorubicin, Vincristine, Etoposide, Prednisone; CHOP, Cyclophosphamide, Doxorubicin, Vincristine, Prednisone; CTCL, cutaneous T-cell lymphoma; EPOCH, Etoposide, Prednisone, Vincristine, Cyclophosphamide, Doxorubicin; IFRT, involved field radiation therapy; LDH, lactate dehydrogenase; LPL, lymphoplasmacytic lymphoma; MGUS, monoclonal gammopathy of undetermined significance; MM, multiple myeloma; PCD, plasma cell dyscrasia; PTCL-NOS, peripheral T-cell lymphoma-not otherwise specified; R-CHOP, Rituximab–Cyclophosphamide, Doxorubicin, Vincristine, Prednisone; R-CVP, Rituximab–Cyclophosphamide, Vincristine, Prednisone; T-LGLL, T-cell large granular lymphocytic leukemia.

### T-LGLL Patients and Treatment Response

Fourteen patients had T-LGLL with the median age at T-LGLL diagnosis of 63 (39–82; SD, 10.1) years, and the median age at PCD diagnosis was 64 (48–82; SD, 9.3) years. Nine patients (64%) were male, and 13 (93%) were Caucasian. Baseline characteristics for these patients are in **Table 2**. Among the T-LGLL patients, eight (57%) had MGUS as their PCD, while four (29%) (n = 4) and two (14%) had MM and plasmacytosis, respectively. At the time of T-LGLL diagnosis, seven patients (50%) presented with anemia [hemoglobin (Hgb) < 12 g/dl], one (7%) presented with neutropenia [absolute neutrophil count (ANC) < 1,500/mm^3^], three (21%) presented with both anemia and neutropenia (two having ANC <500 and one with ANC <1,500), and three (21%) were unknown. Of the four total patients that had neutropenia at presentation, three had severe neutropenia with an ANC <500/mm^3^. Nine patients (64%) were found to have a concomitant autoimmune disease including five (36%) with rheumatoid arthritis and one each (7%) with immune thrombocytopenic purpura, anti-MAG neuropathy, ANCA-associated vasculitis, and cryoglobulinemia. For patients in the T-LGLL cohort, at the time of PCD diagnosis, nine patients (64%) had anemia (Hgb <12 g/dl), and two patients (14%) had bone disease. Six patients (43%) had a serum creatinine (Cr) <1 mg/dl, while six (43%) had a Cr between 1 and 2 mg/dl, one (7%) had a Cr >3 mg/dl, and one (7%) was unknown. No clear preponderance of any particular monoclonal protein-light chain was observed (**Table 2**). Among patients with T-LGLL, 10 (71%) were treated for T-LGLL frontline, while 3 (21%) were treated for their PCD frontline. The most common frontline therapy for T-LGLL was methotrexate n=5 (36%), followed by cyclosporine (CsA) n=3 (21%). One patient (7.1%) received cyclophosphamide (Cy) and one received Cy, Doxorubicin, Vincristine, and Prednisone (CHOP). For patients that had initial treatment for their PCD (n=3), two (14%) received Bortezomib/Lenalidomide/Dexamethasone, and one (7.1%) received Cy/Bortezomib/Dexamethasone (CyBorD). Using strict E5998 criteria for response, the frontline ORR among T-LGLL patients was 2/12 (16.7%), with 8.3% (1/12) with PR and 8.3% (1/12) achieving a CR (**Figure 3**). The median time to response was 2.5 months with a median duration of response of 8.5 months. Five additional patients would go on to have a response (4 PR, 1 CR) with further lines of therapy for an overall response rate of 58% (7/12) for any line of therapy. There were no patients who had clearance of their T-LGLL clone with treatment of their concomitant PCD.

**Table 2:** Baseline characteristics for patients with T-LGLL.

Variable	Total (%) (n=14)
Age at T-LGLL, mean (SD)	62.8 (10.1)
Age at PCD diagnosis, mean (SD)	63.6 (9.3)
***Sex***	
Male	9 (64.3)
Female	5 (35.7)
***Race***	
Caucasian	13 (92.9)
African American	1 (7.1)
***Plasma Cell Dyscrasia***	
MGUS	8 (57.1)
MM	4 (28.6)
Plasmacytosis	2 (14.3)
***Presenting Cytopenia at T-LGLL Diagnosis***	
Neutropenia (ANC **<**1500)	1 (7.1)
Anemia (Hgb **<**12)	7 (50.0)
Both	3 (21.4)
Unknown	3 (21.4)
***TCR V-Beta Positive at T-LGLL Diagnosis***	
Yes	8 (57.1)
No	4 (28.6)
Unknown	2 (14.3)
***LGL Count (CD3CD8+) at Diagnosis***	
<1,500	6 (42.9)
≥1,500	5 (35.7)
Unknown	3 (21.4)
***LDH at T-LGLL Diagnosis***	
≤190	10 (71.4)
>190	3 (21.4)
Unknown	1 (7.1)
***Splenomegaly***	
Yes	4 (28.6)
No	10 (71.4)
***Associated Autoimmune Disease***	
Rheumatoid arthritis	5 (35.7)
ITP	1 (7.1)
Anti-MAG neuropathy	1 (7.1)
ANCA-associated vasculitis	1 (7.1)
Cryoglobulinemia	1 (7.1)
***Anemia (Hgb <12) at PCD Diagnosis***	
Yes	9 (64.3)
No	4 (28.6)
Unknown	1 (7.1)
***Bone Disease at PCD Diagnosis***	
Yes	2 (14.3)
No	6 (42.9)
Unknown	6 (42.9)
***Creatinine at PCD Diagnosis***	
<1.0	6 (42.9)
1.0–1.5	4 (28.6)
1.5–2.0	2 (14.3)
2.0–2.5	0 (0.0)
2.5–3.0	0 (0.0)
>3.0	1 (7.1)
Unknown	1 (7.1)
***Monoclonal Protein-Light Chain***	
IgA-Unk	1 (7.1)
IgG-K	2 (14.3)
IgG-L	3 (21.4)
IgM-K	2 (14.3)
IgM-L	1 (7.1)
N/A-K	1 (7.1)
N/A-L	2 (14.3)
None detected	2 (14.3)
***ISS Staging For PCD***	
1	2 (14.3)
2	1 (7.1)
3	1 (7.1)
N/A	10 (71.4)
***First-Line LGL Therapy***	10/14* (71.4)
Methotrexate	5 (35.7)
Cyclophosphamide	1 (7.1)
Cyclosporine	3 (21.4)
CHOP	1 (7.1)
First-Line PCD Therapy*	3/14* (21.4)
Bortezomib/Lenalidomide/Dexamethasone	2 (14.3)
Cyclophosphamide/Dexamethasone/Bortezomib	1 (7.1)

*One patient has not received treatment for either disease.

### T-Cell Lymphoma Patients and Treatment Response

Twelve patients had TCL with a median age at TCL diagnosis of 64 (range, 41–80; SD, 11.9) years. Eight (67%) of the patients were male, and all of these were Caucasian. Baseline characteristics for these patients are in **Table 3**. Six patients (50%) had PTCL, and six patients (50%) had CTCL. Of the PTCL patients, four had PTCL-NOS and two had AITL. Four (33%) of the patients had MGUS as their PCD, while five (42%) had MM, and one patient (8.3%) had each of plasmacytosis, plasmacytoma, and Kappa light chain-predominant plasma cell proliferation. For patients with PTCL, using Ann Arbor staging, one (16.7%) patient had stage I disease, one (16.7%) had stage II disease, two (33%) had stage III disease, and two (33%) had stage IV disease. For patients with CTCL, four (66.7%) had stage I disease, and one (16.7) patient had stage IV disease, while for one patient, this was unknown. Five patients (42%) had CD30+ disease. At the time of PCD diagnosis, eight patients (67%) had anemia (Hgb <12), and six patients (50%) had bone disease. The most common monoclonal protein-light chain that was seen was immunoglobulin G (IgG)-kappa, seen in six patients (50%). Among patients receiving frontline treatment for their PTCL, the therapies were CHOP (n = 2, 16.7%) and Etoposide, Prednisone, Vincristine, Cyclophosphamide, Doxorubicin (EPOCH) (n = 2, 16.7%). Using Lugano criteria, the ORR to frontline treatment for PTCL was 3/6 (50%), with two (33%) CR and one PR (17%), while three (50%) had progressive disease (**Figure 3**). The median time to response was 4.5 months. For two (16.7%) patients, the initial treatment was for CTCL with skin-directed therapy including one patient receiving topical steroids and one patient receiving bexarotene/extracorporeal photopheresis. Of the six total patients that had CTCL, four received treatment, with an ORR of 75% with 3/4 having a response (2 CR and 1 PR). Two patients were on observation only for their CTCL.

**Table 3:** Baseline characteristics for patients with T-cell lymphoma (TCL).

Variable	Total (%) (n=12)
Age at TCL diagnosis, mean (SD)	63.8 (11.9)
Age at PCD diagnosis, mean (SD)	63.9 (15.1)
***Sex***	
Male	8 (66.7)
Female	4 (33.3)
***Race***	
Caucasian	12 (100.0)
African American	0 (0.0)
***T-Cell Lymphoma***	
PTCL	6 (50.0)
-PTCL-NOS	4 (33.3)
-AITL	2 (16.7)
CTCL	6 (50.0)
***Plasma Cell Dyscrasia***	
MGUS	4 (33.3)
MM	5 (41.7)
Plasmacytosis	1 (8.3)
Plasmacytoma	1 (8.3)
kappa light chain-predominant plasma cell proliferation	1 (8.3)
***Presenting Cytopenia at TCL Diagnosis***	
Neutropenia (ANC <1500)	1 (8.3)
Anemia (Hgb <12)	5 (41.7)
Neither	3 (25.0)
Unknown	3 (25.0)
***Stage at PTCL Diagnosis***	N=6
I	1 (16.7)
II	1 (16.7)
III	2 (33.3)
IV	2 (33.3)
***Stage at CTCL Diagnosis***	N=6
I	1 (16.7)
II	0 (0.0)
III	0 (0.0)
IV	4 (66.7)
Unknown	1 (16.7)
***LDH at TCL Diagnosis***	
≤190	2 (16.7)
>190	6 (50.0)
Unknown	4 (33.3)
***CD30+ at TCL Diagnosis***	
Yes	5 (41.7)
No	3 (25.0)
Unknown	4 (33.3)
***HIV Positive at TCL Diagnosis***	
Yes	0 (0.0)
No	8 (66.7)
Unknown	4 (33.3)
***HTLV-1 Positive at TCL Diagnosis***	
Yes	1 (8.3)
No	3 (25.0)
Unknown	8 (66.7)
***Splenomegaly***	
Yes	1 (8.3)
No	9 (75.0)
Unknown	2 (16.7)
***Associated Autoimmune Disease***	
Autoimmune Hemolytic Anemia	2 (16.7)
None	10 (83.3)
***Anemia (Hgb <12) at PCD Diagnosis***	
Yes	8 (66.7)
No	3 (25.0)
Unknown	1 (8.3)
***Bone Disease at PCD Diagnosis***	
Yes	6 (50.0)
No	4 (33.3)
Unknown	2 (16.7)
***Creatinine at PCD Diagnosis***	
<1.0	6 (50.0)
1.0-1.5	4 (33.3)
1.5-2.0	1 (8.3)
2.0-2.5	0 (0.0)
2.5-3.0	0 (0.0)
>3.0	0 (0.0)
Unknown	1 (8.3)
***Monoclonal Protein-Light Chain***	
IgA-L	1 (8.3)
IgA-Unk	2 (16.7)
IgG-K	6 (50.0)
IgM-L	1 (8.3)
N/A-K	1 (8.3)
Unknown	1 (8.3)
***ISS Staging For PCD***	
1	2 (16.7)
2	1 (8.3)
3	2 (16.7)
N/A	7 (58.3)
***First-Line TCL Therapy***	6/12 (50)
CHOP	2 (16.7)
EPOCH	2 (16.7)
Skin Directed Therapy	2 (16.7)
***First-Line PCD Therapy***	6/12 (50)
Bortezomib/Lenalidomide/Dexamethasone	2 (16.7)
Bortezomib/Dexamethasone	1 (8.3)
Daratumumab/Lenalidomide	1 (8.3)
Docetaxel/Vincristine/Dexamethasone	1 (8.3)
IFRT	1 (8.3)

### Patients Presenting with PCD Frontline

Nine (35%) patients were treated initially for their PCD. Three (33%) patients had T-LGLL, two (22%) had PTCL, and four (44%) had CTCL. Seven (78%) patients had MM, one (11%) patient had MGUS [decision was made to treat this patient with CyBorD due to the patient being in acute renal failure for suspected monoclonal gammopathy of renal significance (MGRS) and when the patient stabilized, and it if was determined that the patient had MGUS, then treatment was stopped), and one (11%) patient had a solitary plasmacytoma. Four (44%) patients were treated with Bortezomib/Lenalidomide/Dexamethasone, and one (11%) patient each was treated with Bortezomib/Dexamethasone, CyBorD, Doxorubicin/Vincristine/Dexamethasone, and Daratumumab/Lenalidomide, and involved field radiation therapy (IFRT) of 50 Gy. Nine patients received frontline treatment for their PCD, with two (22%) achieving VGPR, three (33%) achieving PR, three (33%) achieving SD, and one (11%) with unknown response to frontline therapy. Six patients would go on to receive treatment for their T-cell malignancy, with four (66%) achieving CR, one (17%) achieving PR, and one (17%) with NR. Two patients had high-dose Melphalan with autologous stem cell transplant (HDM-ASCT) after their first line of treatment, and one patient had HDM-ASCT after their second line treatment. Three of the nine (33%) patients in this group would achieve clearance of their PCD clone with T-cell directed therapy, but no patients in the group would achieve clearance of their T-cell clone at any point.

### Clearance of Concomitant PCD Clone in Patients Treated for T-Cell Malignancies

We next evaluated whether patient’s concomitant neoplasm responded to treatment of the primary disease. At our institution, the eradication of the clone is evaluated by bone biopsy with aspirate and protein electrophoresis/free light chain assay in the serum or the urine of the patients. This is in accordance with IMWG criteria. None of our patients had MRD assessment, which was performed by ClonoSEQ assay (Adaptive Biotechnologies Corporation, Seattle, USA), and none were evaluated with high-sensitivity flow cytometry. Within the entire cohort (n=26), 8/26 had clearance of their PCD clone. Of these patients, four were treated for both diseases, three were treated for only their T-cell malignancy, and one was treated for only their PCD. Full breakdown can be seen in **Table 5**. Of the patients who received treatment for their T-cell malignancy frontline, 31.3% (5/16) patients had clearance of their PCD clone.

Four (50%) of the patients had their clone clear after starting treatment for their T-cell malignancy, including two (25%) who never received PCD-directed therapy. The treatments included Cy (one patient), Bexarotene (one patient), and MTX (two patients; one with prednisone and one without prednisone). An additional patient has an unknown initial T-LGLL treatment date, but they were on CsA (for kidney transplant), a known T-LGLL treatment, at the time of the resolution of their PCD clone. Of the patients who received initial frontline treatment for their PCD, 33.3% (3/9) had clearance of their PCD clone. This included two patients with clearance after treatment for MM and one after treatment for a plasmacytoma. The treatments leading to resolution included Azacitidine/Bortezomib/Dexamethasone for MM (and MDS); Bortezomib, Lenalidomide/Dexamethasone for MM; and Bortezomib/Dexamethasone for plasmacytoma.

Of patients who received treatment for their T-LGLL, 41.7% (5/12) had clearance of their PCD clone, and neither of the two patients that were on observation for their T-LGLL had clearance of their PCD clone. No patient had clearance of their T-cell clone due to treatment of their PCD.

### Survival Outcomes

**Figure 1:**
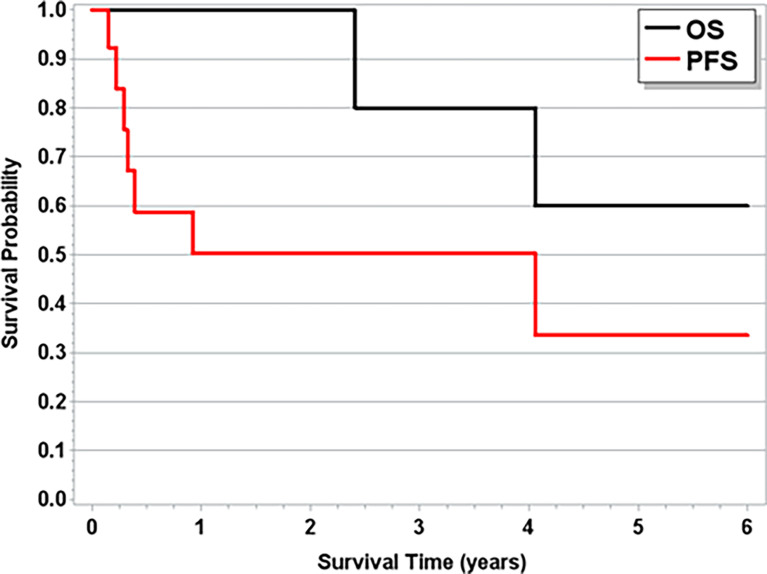
Overall Survival and Progression Free Survival for Entire Cohort.

**Figure 2:**
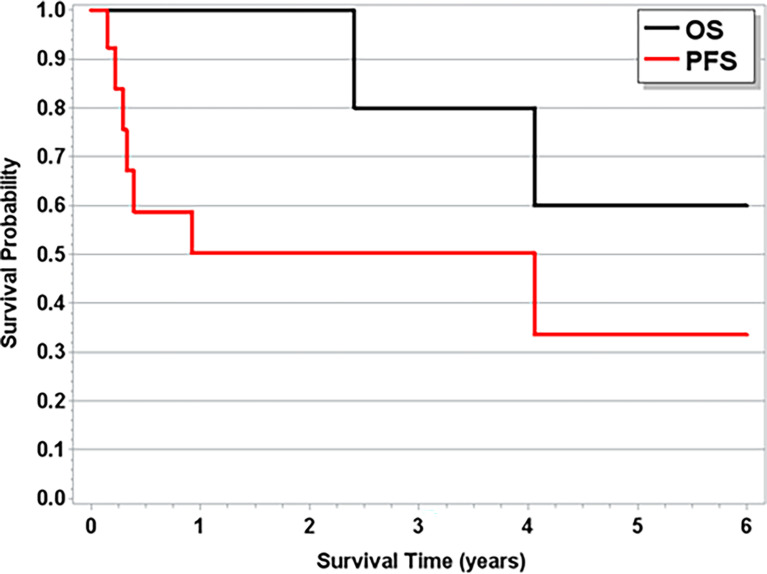
Overall Survival and Progression Free Survival for Patients with T-LGLL.

**Figure 3:**
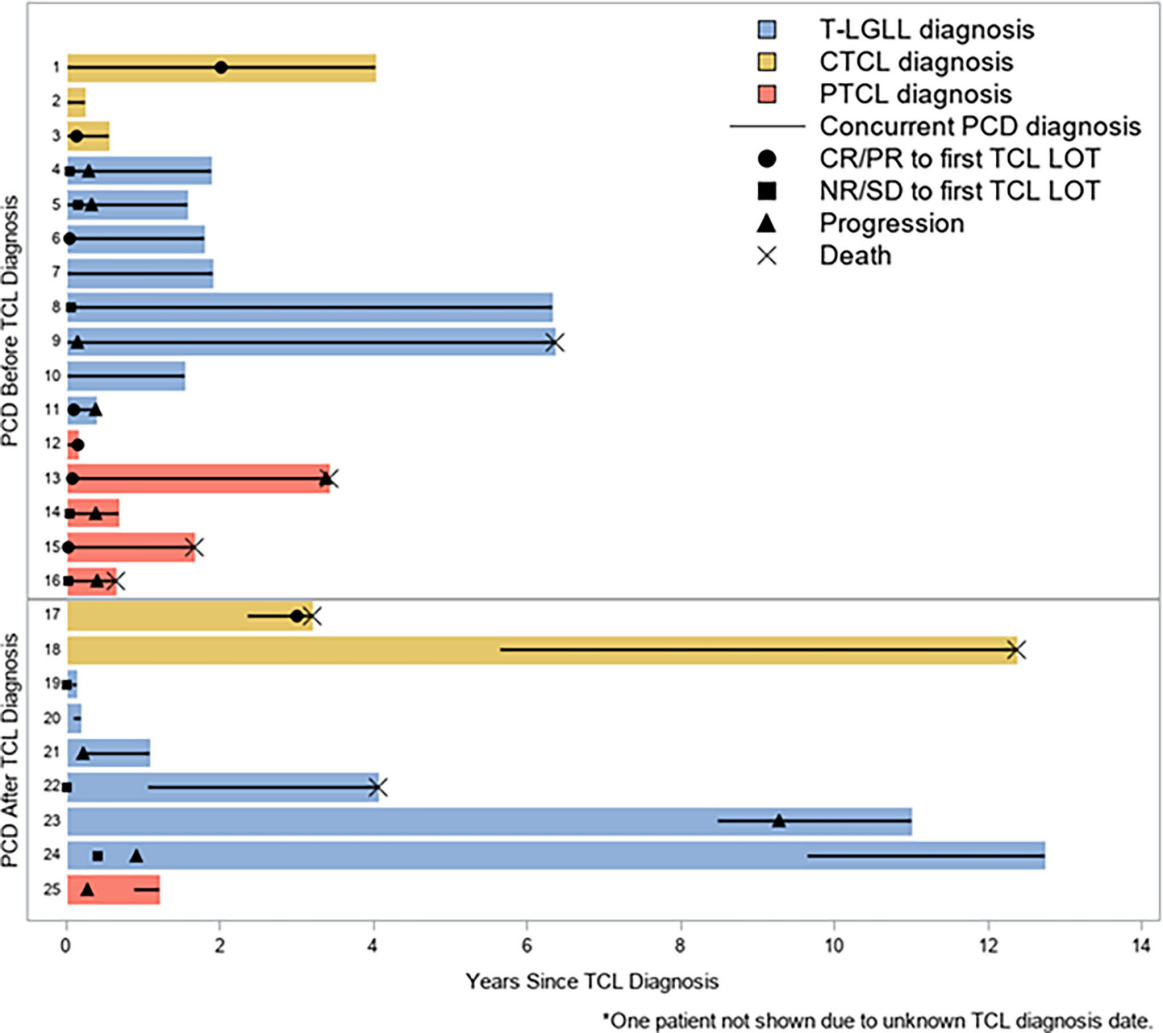
Swimmer's Plot for Entire Cohort. Swimmer's Plot showing all patients in relation of time of diagnosis of T-Cell Lymphoma (TCL). Patients are split by whether they were diagnosed with T-cell Malignancy or PCD first. Lines on the solid color bars represent concurrent diagnosis. Legend describes when patients had progression or death.

With a median follow-up time of 1.8 years (range, 3 weeks–12.8 years), the median OS across all patients was 4.1 years (**Figure 1**). The median follow-up time for patients with T-LGLL was 1.9 years (range, 7 weeks–12.7 years), and for patients with TCL, it was 1.21 years (3 weeks–12.4 years). For full progression and survival outcomes, see **Tables 4A**, **B**. The median OS for patients with T-LGLL was not reached (**Figure 2**), while the median OS for patients with TCL was 3.4 years (**Supplementary Figure S1**). When TCL is broken down by disease, the median OS for PTCL was 1.7 years, and the median OS for CTCL was 12.4 years. In total, 42.3% of patients had progression of their T-cell malignancy. Six of the 12 (50%) patients with T-LGLL and 4/6 (67%) of patients with PTCL had refractory disease, while 0% with CTCL had progression (on frontline treatment). Median overall PFS was 3.21 years. For patients with T-LGLL, the median leukemia-free survival was 11 months (**Figure 2**), and for patients with TCL, the median PFS was 3.21 years (**Supplementary Figure S1**). When broken down by type of TCL (PTCL or CTCL, the median PFS among CTCL patients was 12.37 years, and the median PFS for PTCL patients was only 4.8 months. Full progression and response per patient are seen in **Figure 3** with treatment regimens in **Supplementary Table S2**. Of the patients who received treatment for their T-cell malignancy, 40% (8/20) had a response (3 PR and 5 CR). Of the patients who received treatment for their PCD, 60% (6/10) had a response (3 PR and 3 VGPR). For patients who had MM (n=8), the median PFS was 3.4 years, and the OS was 7.9 years. Full response rates by disease are seen in **Supplementary Table S1**.

**Table 4A:** Progression and survival outcomes.

Outcome	All	T-Cell Lymphoma	T-LGLL
Progression	11/26 (42.3%)	4/12 (33.3%)	7/14 (50.0%)
Death	7/26 (26.9%)	5/12 (41.7%)	2/14 (14.3%)
Progression or death	15/26 (57.7%)	7/12 (58.3%)	8/14 (57.1%)
Median OS years (95% CI)*	4.06 (2.41-NR)	3.43 (0.65-NR)	NR (2.41-NR)
Median PFS years (95% CI)*	3.21 (0.38-9.28)	3.21 (0.28-NR)	0.92 (0.22-NR)

*One T-cell lymphoma patient was excluded from time-to-event statistics due to unknown diagnosis date.

**Table 4B:** Progression and survival outcomes.

Outcome	All	PTCL	CTCL	T-LGLL
Progression	11/26 (42.3%)	4/6 (66.7%)	0/6 (0%)	7/14 (50.0%)
Death	7/26 (26.9%)	3/6 (50.0%)	2/6 (33.3%)	2/14 (14.3%)
Progression or death	15/26 (57.7%)	5/6 (83.3%)	2/6 (33.3%)	8/14 (57.1%)
Median OS years (95% CI)*	4.06 (2.41-NR)	1.66 (0.65-NR)	12.37 (3.21-NR)	NR (2.41-NR)
Median PFS years (95% CI)*	3.21 (0.38-9.28)	0.40 (0.28-NR)	12.37 (3.21-NR)	0.92 (0.22-NR)

*One CTCL patient excluded from time-to-event statistics due to unknown diagnosis date.

## Discussion

In the present study, we present the largest cohort of patients with concomitant T-cell malignancies and PCD to date, with a focus on survival and treatment outcomes. For the first time, we present treatment response and survival outcomes and demonstrate that treatment of the underlying T-cell malignancy can also eradicate the concomitant PCD clone, which has implications into the pathogenesis of these diseases.

It is important to compare the results observed in this study with the established long-term survival literature for each individual disease. While an imperfect comparison, this helps to provide important, initial insights into the prognostic impact of concomitant PCD with T-cell malignancies. In the patients with T-LGLL in our cohort, the median PFS was 11 months, and OS was not reached (**Figure 2**). The OS is consistent with the established literature, as patients with T-LGLL are known to have a prolonged OS, with the ECOG 5998 study also having an OS not reached and Braunstein et al. showed a 5-year OS of 72% (4, 20, 30). Among CTCL patients, the observed median PFS of 12.4 years is similar to expected survival rates previously published for CTCL (31). Based upon our results, for patients with CTCL and T-LGLL, the survival outcomes are as expected per published literature for the respective disease types, suggesting that these patients should be treated for the first diagnosed, underlying disorder. The six patients with PTCL had a median OS of 1.7 years and a median PFS of 4.8 months. All of these patients were newly diagnosed patients with IPI scores ranging from 0 to 4. In the paper by Vose et al., median OS was nearly 2.5 years for PTCL-NOS, and AITL showed a median OS of approximately 2.2 years. The results in our series among PTCL patients are worse than expected/known outcomes for these lymphomas, suggesting that patients with a concomitant PCD may have more aggressive or chemo-resistant disease (**Figure 3**). The exact reason why these patients may be experiencing worse outcomes is not known. Of the six patients with PTCL, two had AITL, and four were PTCL-NOS. AITL is a lymphoma of T-follicular helper (TFH)-derived T-lymphocytes, and over the past 10 years, some patients with previously unclassified PTCL (PTCL-NOS) have been reclassified as TFH under 2016 WHO guidelines (32). These patients often present with inflammatory symptoms (skin rash, edema, and arthralgias) c/w the B-cell regulatory function of these cells. Furthermore, it is likely that patients who have lymphomas derived from TFH cells are more likely to have concomitant PCD, as they are inherent malignancies of regulatory T-cells, and in these cases, the T-cell process likely drives the PCD (33). Frequently, these patients have complex pathological characteristics, and with the concomitant PCD, diagnosis is often protracted and delayed, which may delay treatment initiation. This highlights the importance of considering T-cell malignancies in the differential for patients with atypical PCD. While our population of PTCL patients is small (n=6), this concerning trend will need to be evaluated in a larger population of patients with additional studies for confirmation and suggests that aggressive treatment is needed for this population. Finally, we also observed patients who had resolution of their PCD clone after being treated with only T-cell-directed therapy (two patients with T-LGLL and one patient with CTCL). Furthermore, two patients had resolution of their PCD clone only after starting treatment for their T-cell malignancy (one patient with a CR for MM and one patient with resolution of their plasmacytosis; both had T-LGLL) (**Table 5**). This is an important finding, as it shows that the T-cell malignancy may be driving the monoclonal plasma cell spike and suggests that the underlying pathophysiology may be driven by the T-cell process. There is support that T-regulatory cells may maintain plasma cells, but the exact mechanism is unknown (34).

**Table 5:** Patients with clearance of PCD clone.

Patient Number	T-Cell Malignancy	T-cell treatment or PCD treatment first?*	First Line T-Cell Treatment	T-Cell Progression?	PCD	First Line PCD Treatment	PCD Progression After First Line Treatment?	PCD Clearance after T-Cell Treatment?
3	T-LGLL	Only T-cell	MTX	Yes	Plasmacytosis	None	No	Undetermined*
4	PTCL	PCD	CHOEP	No	Plasmacytoma	IFRT	Yes	No
5	T-LGLL	T-cell	Cyclosporine	No	MM	Bortezomib/Lenalidomide/Dexamethasone	Yes	Yes
11	T-LGLL	T-cell	Methotrexate	No	MM	Cyclophosphamide/Dexamethasone	Yes	Yes
13	T-LGLL	PCD	Cyclophosphamide	No	MM	Bortezomib/Lenalidomide/Dexamethasone	No	No
15	T-LGLL	Only T-cell	Cyclophosphamide	Yes	MGUS	None	No	Yes
18	CTCL	Only PCD	None	No	MM	Daratumumab/Lenalidomide	Yes	No
24	CTCL	Only T-cell	Bexarotene/Extracorporeal Photopheresis	No	MGUS	None	No	Yes

Frontline treatment information for patients that had clearance of their PCD cline and whether they received initial treatment for their T-Cell disease or PCD and whether they had progression to front line treatments.

*Exact start date for T-cell malignancy is unknown, but the patient was on Cyclosporine (Known T-LGLL treatment) at the time of PCD clone clearance.

T-LGLL patients represented the largest type of T-cell malignancy in our series with 14/26 (54%) of patients with T-LGLL. Only 50% of patients with T-LGLL had progression of their disease, and only 14% died. The median OS was not reached in this group, suggesting that there is no deleterious effect of the concomitant PCD process in these patients. Interestingly, 36% of T-LGLL patients in this population had eradication of their plasma cell clone with T-LGLL directed treatment, including three patients with MM whose PCD clone was not fully eradicated with frontline myeloma-directed therapy but resolved after T-LGLL-directed treatment. This provides further evidence that the T-cell process may be driving the PCD, and treatment of the underlying T-cell malignancy, especially T-LGLL, can potentiate the eradication of the PCD clone. It has been suggested that treating the PCD clone may suppress the T-LGLL clone (16), but in our cohort, 38% of the patients who had eventual eradication of their PCD clone had treatment only for their T-cell malignancy. This does make rational sense, as patients who received T-cell-directed therapies often receive therapeutics that are known to be effective against PCD, such as cyclophosphamide. Sidiqui et al. described patients with concurrent T-LGLL and PCD, and in their study, a majority (82%) of patients developed T-LGLL after their PCD or concurrently, whereas in our study, a majority (58%) were diagnosed with their T-cell malignancy first or both malignancies at the same time (16). The variability between these two studies could simply be due to the limited sample size in both studies or earlier detection of the T-LGLL in the present series. Whatever the explanation, further studies are needed to verify the relationship between these two diseases.

It has been hypothesized that B-cell expansion can potentially result due to B-cell dysfunction in the setting of T-LGLL (35), and this relationship has been seen with AITL and plasma cell proliferation as well (36). We show for the first time that treating the patient’s T-cell malignancy may eradicate the PCD clone, especially if the patient has T-LGLL. We even see eradication of the plasma cell clone in 50% of patients with MM in this cohort. The T-LGLL may be driving the expansion of B cells as described above, leading to the development of a plasma cell clone. When the T-LGLL is treated, this clonal expansion resolves. It remains unknown whether the PCD drives the T-cell disorder or *vice versa*. To date, the exact pathophysiological mechanisms of concurrent PCD and T-cell malignancy are unknown. In MM, about one-third of patients can develop TCR-β rearrangements that share a similar immunophenotype to T-LGLL (37). Furthermore, given that T-LGLL is a disorder of terminal effector T-lymphocytes, it is possible that this induces the development of a reactive clonal expansion due to the underlying PCD or monoclonal gammopathy (38). This could be from an enhanced clonal expansion due to the chronic immune response that was initially due to the PCD (39).

This study has limitations that are inherent to all retrospective, single-center studies. The study encompassed a long period of time, during which treatment strategies changed and new agents became available. Additionally, analysis of clinical outcomes to treatment must be interpreted with caution, given low patient numbers, and only analyzing for initial progression or death. Furthermore, due to the multiple different diseases, the first-line treatment for the patients in this cohort varied extensively. It is difficult to correlate clearance of the PCD clone with survival, as only a small portion of patients had their clone resolve; it was nearly evenly split between patients who received treatment for both their T-cell malignancy and their PCD, or just treatment for the T-cell malignancy. Despite these limitations inherent to retrospective analyses, this study provides the largest dataset of patients with concomitant T-cell malignancies and PCD to date, providing a robust insight into this likely underdiagnosed population. A large multicenter retrospective review is needed to further characterize this population and definitively identify the clinical significance of these concomitant disorders. We show that treating the patient’s T-cell malignancy has similar OS and PFS as compared to established baselines for T-LGLL and CTCL and may even have the potential to eradicate the PCD clone. However, for patients with PTCL (PTCL-NOS and AITL), outcomes appear worse, with similar ORR, but worse PFS, suggesting that the presence of a concomitant PCD may increase the overall risk in these patients.

## Conclusion

We present the largest study to date on patients who have concomitant T-cell malignancies and plasma cell dyscrasias. In our analysis, we found that there was no survival difference in patients that have concomitant CTCL and T-LGLL and PCD when treated with standard T-cell-directed therapy. However, patients with concomitant PCD and PTCL had significantly inferior outcomes, with rapid progression, and worse OS and PFS highlighting the need to further evaluate these patients in a large, multi-center setting. For patients with T-cell malignancies as the primary diagnosis with concomitant PCD, treatment with standard T-cell-directed therapies is recommended with continued follow-up and monitoring of the concomitant PCD. There is the potential that treating a patient’s T-cell malignancy may lead to resolution of their PCD clone, even without therapy directed at the PCD. Larger, multi-center studies are needed to validate these findings, and definitively describe the effect of concomitant T-cell malignancies and PCD.

## Data Availability Statement

The original contributions presented in the study are included in the article/**Supplementary Material**. Further inquiries can be directed to the corresponding author.

## Ethics Statement

The studies involving human participants were reviewed and approved by the Ohio State University Wexner Medical Center IRB. Written informed consent for participation was not required for this study in accordance with the national legislation and the institutional requirements.

## Author Contributions

ZB and MR collected the data. ZB, MR, AER, and JEB analyzed the data and wrote the manuscript. EM and LW performed the statistical analysis. ZB, AMi, AER, and JEB designed the study. ZB, MR, NB, DB, SD, MC, AK, FC, WH, RB, CC, DA, NC, AMe, WJ, PP, JCR, AER, and JEB cared for the patients. All authors contributed to the article and approved the submitted version.

## Funding

This study was supported by NIH/NCATS KL2TR002734 (to JEB). DA is supported by NIH grant number K23-HL155890, and an American Heart Association‐Robert Wood Johnson Foundation (Harold Amos) Program grant.

## Conflict of Interest

The authors declare that the research was conducted in the absence of any commercial or financial relationships that could be construed as a potential conflict of interest.

## Publisher’s Note

All claims expressed in this article are solely those of the authors and do not necessarily represent those of their affiliated organizations, or those of the publisher, the editors and the reviewers. Any product that may be evaluated in this article, or claim that may be made by its manufacturer, is not guaranteed or endorsed by the publisher.
