# Supplementary figures and images for: Incidence, Treatment, and Survival of Patients With T-Cell Lymphoma, T-Cell Large Granular Leukemia, and Concomitant Plasma Cell Dyscrasias
Source: Front Oncol. 2022 Apr 29;12:858426. doi: 10.3389/fonc.2022.858426 (PMC9106372; doi:10.3389/fonc.2022.858426)

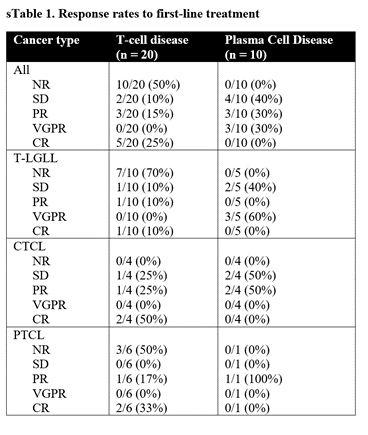

Supplement: Supplementary file 1 [file DataSheet_1.zip › sTable 1.PNG]

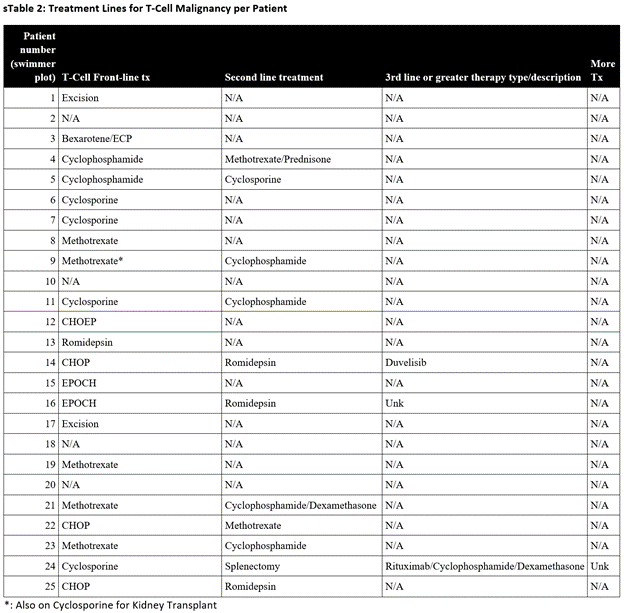

Supplement: Supplementary file 1 [file DataSheet_1.zip › sTable 2.PNG]

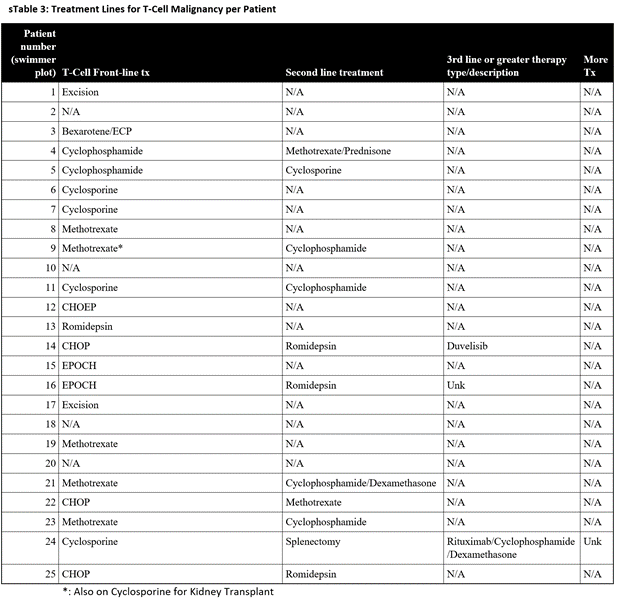

Supplement: Supplementary file 1 [file DataSheet_1.zip › sTable 3.PNG]

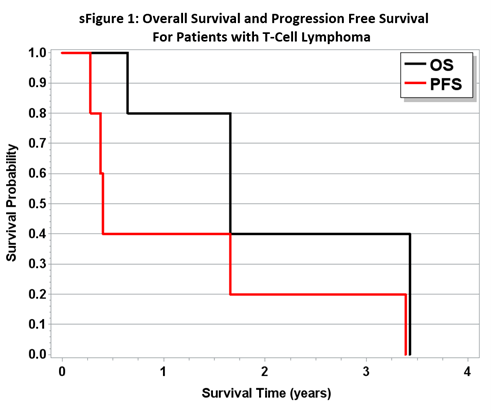

Supplement: Supplementary file 2 [file Image_1.png]
